# Prevalence of amniotomy in Sweden: a nationwide register study

**DOI:** 10.1186/s12884-022-04805-w

**Published:** 2022-06-14

**Authors:** Sofia Tallhage, Kristofer Årestedt, Kristina Schildmeijer, Marie Oscarsson

**Affiliations:** 1 Faculty of Health and Life Sciences, Linnaeus University, 391 82 Kalmar, Sweden; 2 Department of Obstetrics and Gynecology, Region Kalmar County, 392 44 Kalmar, Sweden; 3 Department of Research, Region Kalmar County, 392 44 Kalmar, Sweden

**Keywords:** Amniotomy, Labor intervention, Nulliparity, Multiparity, Register study, Prevalence

## Abstract

**Background:**

Amniotomy is a commonly used labor intervention with uncertain evidence, and there are complications connected to the intervention. Yet, the Swedish prevalence of amniotomy is unknown. The aim of the study was therefore to describe the prevalence of amniotomy in Sweden.

**Methods:**

This nationwide register-based study included 330,913 women giving birth in 2017–2020. Data were collected from the Swedish Pregnancy Register in which the majority of data is collected via direct transfer from medical records. Prevalence of amniotomy was described for all births, for nulliparous and multiparous women with spontaneous onset of labour, and at the hospital level. Descriptive statistics and chi-square test were used to analyse the data.

**Results:**

For all births, the prevalence of amniotomy was 40.6%. More amniotomies were performed in Robson group 1 compared to Robson group 3; 41.1% vs 32.3% (*p* < 0.001). The prevalence for all births remained the same during the study period; however, a decrease from 37.5 to 34.1%, was seen in Robson group 1 and Robson group 3 (*p* < 0.001). Variations in the prevalence between hospitals were reported. The hospitals with the fewest number of births annually had the highest prevalence of amniotomy (45.0%), and the lowest prevalence was reported at the University hospitals (40.4%) (*p* < 0.001).

**Conclusions:**

Amniotomy is a common labor intervention in Sweden, given that almost half of the laboring women underwent the intervention. Our results, regarding variations in the prevalence between hospitals, could imply a potential for fewer amniotomies in Swedish childbirth care.

## Background

Globally, amniotomy is one of the most used labor interventions with a long tradition in obstetric care [1]. There is a limited number of studies describing the prevalence of amniotomy and those that exist describe prevalence for small population groups [2, 3]. Only one nationwide register study exists, from the Netherlands, in which the prevalence of amniotomy was 46.9% for nulliparous and 57.3% for multiparous women, with single births after 37 weeks of gestation [4]. Labor interventions can be necessary to prevent maternal and perinatal mortality and morbidity [5]. However, over the last two decades, there has been a substantial increase in the use of labor interventions to initiate, monitor, accelerate, or terminate the physiological process of labor. Healthy, low-risk women may be exposed to unnecessary interventions that interfere with the physiological process of childbirth [6]. When labor interventions are overused or used inappropriately, a process referred to as “too much, too soon,” they pose a risk of iatrogenic harm in women and their babies [7]. Furthermore, overuse of interventions leads to unnecessarily high healthcare costs [8]. The global concern regarding an increased use of labor interventions in low-risk populations is a controversial issue of current midwifery and obstetric literature [2, 8–15]. The World Health Organization (WHO) highlights the importance of positive birth experiences and advise using labor interventions only when indicated, and not routinely [5–7].

Amniotomy is used for various reasons, whereof the main purpose is to increase the strength and effectiveness of uterine contractions, and thereby shorten the duration of labor. Amniotomy is, therefore, a routine intervention to induce labor and to treat labor dystocia [1]. It is thought to act by releasing prostaglandins and increasing oxytocin levels and thereby enhancing labor contractions. However, the evidence of amniotomy accelerating labor progress, in spontaneous labor, is weak [1, 6]. Additionally, there are complications connected to the intervention, such as umbilical cord prolapse, vasa previa, ascending infection, and cardiotocography (CTG) abnormalities [1]. There is need for more research to evaluate the appropriate use of interventions in childbirth care, including amniotomy [7]. The evidence for amniotomy in spontaneous labor is weak, and it can cause complications [1], yet, to our best knowledge there is only one nationwide study describing its prevalence [4]. More research is needed to investigate the appropriate prevalence of amniotomy [1]. Information on its prevalence offers opportunities for comparisons and for clinical evaluation. The aim of this study, therefore, was to describe the prevalence of amniotomy in Sweden, with a focus on women with spontaneous onset of labor.

## Methods

This nationwide register-based study was based on data from the Swedish Pregnancy Register. The register was started in 2013; from 2017 to 2020, it included approximately 92% of all births in Sweden. The register contains detailed information on the pregnant women, entered into the electronic medical records by midwives and physicians in a standardized way at antenatal-, delivery-, and postnatal care. The majority of data is collected via direct transfer from the electronic medical records; however, data on antenatal care are web-entered manually by the midwives. The direct electronically transferred variables have been validated with 98–100% agreement with medical records. The web-entered data by antenatal care midwives have been validated with good or very good agreement (≥95%), with medical records [16, 17].

The birth rate in Sweden is approximately 115,000 per year and almost all women give birth in hospitals, a service provided by the state-driven healthcare. In Sweden, midwives are the primary caregivers for intrapartum care when pregnancies and births are healthy, without medical complications. If complications occur during labour, midwives work in collaboration with obstetricians.

**Fig. 1:**
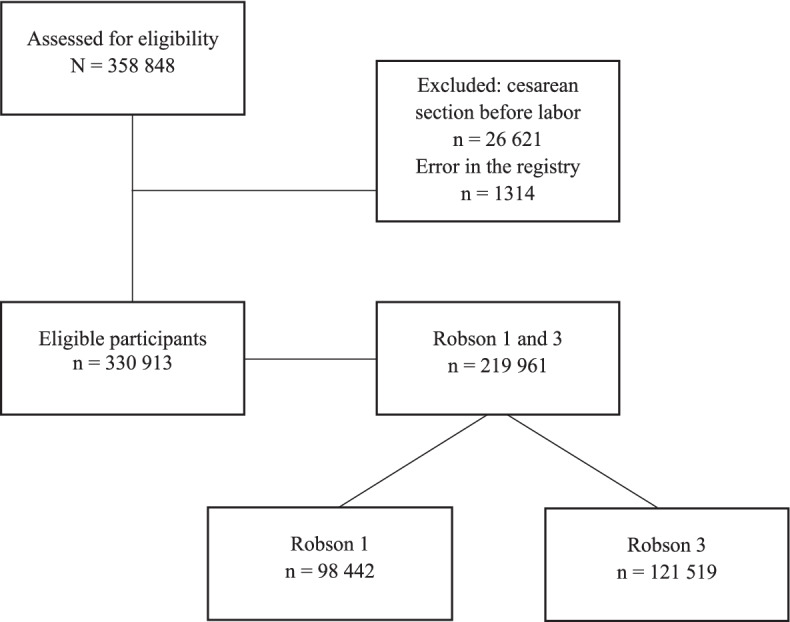
Flowchart of the study population

The study population comprised all women giving birth from January 2017 to June 2020 and were included in the registry (*n* = 358,848). Women with pre-labor cesarean section (*n* = 26,621) were excluded. During the data management, an error in the register was identified in the data set by values on amniotomy and spontaneous rupture of the membranes, being identical and inaccurate. The error was reported to the Pregnancy Register and confirmed. The error was caused by old versions of the medical record system. Subsequently, women with this error (*n* = 1314) were excluded. The Robson classification system was used to identify women with a spontaneous onset of labor, making comparison of the prevalence of amniotomy within groups more clinically relevant [18]. The Robson system classifies all deliveries into one of ten groups on the basis of five parameters: obstetric history, onset of labor, fetal lie, number of neonates, and gestational age [19]. Robson groups 1 and 3 were included. Robson group 1 includes nulliparous women with a single baby with cephalic presentation, at term, and spontaneous onset of labor, *n* = 98,442 (29.7%). Robson group 3 includes multiparous women with a single baby with cephalic presentation, at term, and spontaneous onset of labor, *n* = 121,519 (36.7%) (Fig. 1).

The variables used in the present study included: maternal country of birth, Body Mass Index (BMI) at first antenatal visit, level of education, age at childbirth, Robson Group classification, multiple pregnancy, and mode of birth. Information on the variables, spontaneous rupture of the membranes and amniotomy, were given as date and time if it had occurred. Maternal country of birth was categorized as born in Sweden, born outside of Sweden but in the EU, and born outside of the EU. The BMI was categorized according to the WHO standards into: underweight (< 18.5), normal (18.5–24.9), overweight (25.0–29.9), obesity Class I (30.0–34.9), obesity Class II (35.0–39.9), and obesity Class III (≥40.0) [20]. The included hospitals (*n* = 38) were categorized into five categories, according to the annual birth volumes or profiles, in order to make a hospital level comparison possible [21]. Category one included hospitals across the country with annual birth volumes < 1000 (*n* = 6). Hospital categories two (*n* = 7) and three (*n* = 9) included hospitals with annual birth volumes from 1000 to 1999 and from 2000 to 2999, respectively. The hospital category four included eight hospitals with ≥3000 births per year. University hospitals (*n* = 8) were categorized as its own category, category five, due to the profile of tertiary level care and a different patient mix compared to the other hospitals.

### Statistical analysis

Means and standard deviations were used to present continuous variables, while categorical data were presented as absolute and relative frequencies. As many women had more than one birth during the study period, the statistical assumption of independence could be violated. Therefore, this assumption was tested using an intraclass correlation coefficient (ICC) that compares within-group variance to between group variance. Consequently, a high ICC indicate problems with nonindependence [22]. The ICC was low (0.127) and the births was therefore treated as independent in the statistical analyses. Prevalence of amniotomy was calculated for each hospital and then stratified according to the hospitals’ annual number of births. University hospitals were treated as a separate group, independent of the number of births [21]. For year 2020, prevalence data were only available for January to June. To make it possible to compare the numbers of amniotomy over time, including 2020, the annual prevalence for this year was estimated by multiplying the prevalence from January to June with two. This has not affected the presentation of the prevalence. Pearson’s chi-square test was used to compare the prevalence of amniotomy over time (2017–2020) and between hospitals. In addition to the overall effect, pairwise comparisons were also conducted as post-hoc tests. The statistical significance was overall set at a *p* < 0.05. The Bonferroni corrected *p*-values in the post hoc test were < 0.008 for time and < 0.005 for hospitals. The analyses were carried out using Stata IC 16.0 (StataCorp LLC, College Station, TX, U.S.A.).

## Results

From January 2017 to June 2020, there were 330,913 births, in total. The mean maternal age was 30.9 (SD = 4.9) years, and the mean BMI was 25.1 (SD = 4.9). About two-thirds (*n* = 215,130, 70.5%) of the women were born in Sweden and a fifth (*n* = 63,690, 20.9%) outside of the EU. The majority of women had upper secondary school or university as the highest level of education (*n* = 255,250, 91.2%). Most women (*n* = 273,084, 84.8%) had a spontaneous vaginal birth. In total, 4049 (1.2%) women gave birth to twins. Maternal characteristics on all women, Robson group 1 and Robson group 3, together and respectively, are reported in Table 1.

**Table 1:** Characteristics of the participants

	All births *n* = 330,913	Robson 1 and 3 *n* = 219,961	Robson 1 *n* = 98,442	Robson 3 *n* = 121,519
Age (years), mean (SD)	30.9 (4.9)	30.6 (4.8)	29.0 (4.6)	31.9 (4.6)
BMI (kg/m^2^), mean (SD)	25.1 (4.9)	24.6 (4.5)	24.2 (4.4)	24.9 (4.7)
BMI categorization, n (%)				
< 18.5, n (%)	7967 (2.6)	5880 (2.9)	2968 (3.3)	2912 (2.6)
18.5–24.9	171,406 (55.8)	121,853 (59.6)	57,203 (63.0)	64,650 (56.9)
25–29.9	81,994 (26.7)	51,804 (25.4)	21,427 (23.6)	30,377 (26.8)
30–34.9	31,764 (10.3)	17,913 (8.8)	6645 (7.3)	11,268 (9.9)
35–39.9	10,456 (3.4)	5213 (2.5)	1914 (2.1)	3299 (2.9)
> 40	3715 (1.2)	1643 (0.8)	614 (0.7)	1029 (0.9)
Unknown	23,611	15,665	7671	7984
Country of origin, n (%)				
Sweden	215,130 (70.5)	144,286 (70.9)	68,089 (75.0)	76,197 (67.5)
Born outside Sweden but in the EU	26,093 (8.6)	17,871 (8.8)	7816 (8.6)	10,055 (8.9)
Born outside the EU	63,690 (20.9)	41,429 (20.3)	14,860 (16.4)	26,569 (23.5)
Unknown	26,000	16,375	7677	8698
Highest level of education, n (%)				
No education or education < 9 years	17,420 (6.2)	11,204 (6.0)	4044 (4.8)	7160 (6.9)
Lower secondary school	7320 (2.6)	4686 (2.5)	1008 (1.2)	3678 (3.6)
Upper secondary school	149,834 (53.5)	101,849 (54.3)	48,237 (57.1)	53,612 (52.0)
University	105,416 (37.6)	69,707 (37.2)	31,124 (36.9)	38,583 (37.4)
Unknown	50,923	32,515	14,029	18,486
Multiple pregnancies, n (%)	4089 (1.2)	–	–	–
Mode of birth, n (%)				
Spontaneously vaginal	273,084 (84.8)	198,420 (90.6)	80,904 (82.4)	117,516 (97.2)
Instrumental	17,676 (5.5)	11,183 (5.1)	9742 (9.9)	1441 (1.2)
Emergency cesarean section	31,166 (9.7)	9504 (4.3)	7586 (7.7)	1918 (1.6)
Unknown	8987	854	210	644

Of the 330,913 births, amniotomy was performed in 134,493 (40.6%). A total of 184,966 (55.9%) women had spontaneous rupture of the membranes. The prevalence of amniotomy for Robson groups 1 and 3 together was 36.2%; however, when separated, the prevalence was higher for Robson group 1 compared to Robson group 3, 41.1% vs 32.3% (*p* < 0.001).

**Fig. 2:**
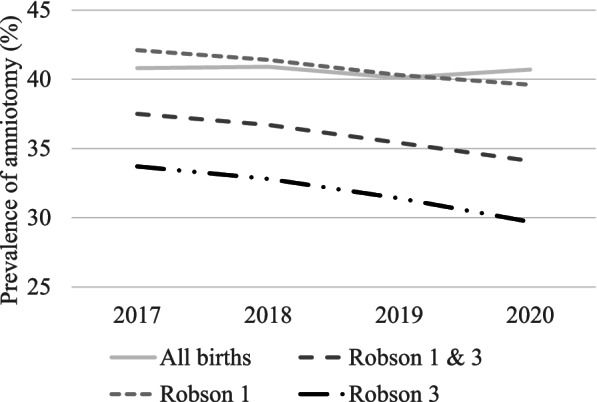
Prevalence of amniotomy each year of the study period

For all births, there were no differences in the prevalence over the study period (*p* = 0.678). However, for Robson group 1 and Robson group 3, the prevalence of amniotomy decreased from 37.5 to 34.1% (*p* < 0.001). The same pattern was also shown in the separate analyses of Robson 1 and Robson 3 (Fig. 2). However, in the post hoc analyses of Robson group 1, no significant difference was detected between 2017 and 2018 and between 2019 and 2020. For the Robson group 3, no differences were detected between 2017 and 2018 (Table 2).

**Table 2:** Number of births and prevalence of amniotomy between 2017-2020^a^

		2017	2018	2019	2020^a^	*p*-value^b^	Post-hoc test^c^
All births,	Number of births, n	94,642	94,958	93,604	47,709		
*n* = 330,913	Amniotomy, n (%)	38,612 (40.8)	38,880 (40.9)	37,560 (40.1)	19,441 (40.7)	0.678	
Robson group 1 and 3,	Number of births, n	64,615	64,042	61,528	29,776		
*n* = 219,961	Amniotomy, n (%)	24,200 (37.5)	23,518 (36.7)	21,788 (35.4)	10,147 (34.1)	< 0.001	ABCDEF
Robson group 1,	Number of births, n	28,888	28,935	27,529	13,090		
*n* = 98,442	Amniotomy, n (%)	12,163 (42.1)	11,989 (41.4)	11,099 (40.3)	5186 (39.6)	< 0.001	-BCDE-
Robson group 3,	Number of births, n	35,727	35,107	33,999	16,686		
*n* = 121,519	Amniotomy, n (%)	12,037 (33.7)	11,529 (32.8)	10,689 (31.4)	4961 (29.7)	< 0.001	-BCDEF

^a^ 1 January to 30 June, 2020

^b^ Pearson chi-square test

^c^ Pairwise comparisons using the Pearson chi-square test with Bonferroni corrected *p*-values (< 0.008); significant differences are presented as: A = 2017 ≠ 2018, B = 2017 ≠ 2019, C = 2017 ≠ 2020, D = 2018 ≠ 2019, E = 2018 ≠ 2020, F = 2019 ≠ 2020,

Variations in the prevalence of amniotomy between hospitals were seen. For all births, the prevalence of amniotomy was highest at the hospitals with ≤1000 births annually and the lowest at hospitals with > 2000 births annually and at University hospitals. For Robson groups 1 and 3, the prevalence was generally lower; however, the pattern was similar. The hospitals with ≤1000 births annually had a higher prevalence than hospitals with more births. The University hospitals had the lowest prevalence in this group (*p* < 0.001). The same pattern was seen in the separate analysis of Robson group 1 and Robson group 3. For Robson group 1, the prevalence of amniotomy ranged from 33.6% at the hospital with the lowest prevalence to 61.8% at the hospital with the highest prevalence (Table 3).

**Table 3:** Number of births and prevalence of amniotomy in different hospital categories between 1 January 2017 and 30 June 2020

				Prevalence, %		
	Hospital category	Number of hospitals, *n* = 38	Number of births, n	Total^a^, (lowest-highest)	*p*-value^b^	Post-hoc test^c^
All births *n* = 330,913	< 1000 births annually	6	14,020	45.0 (39.3–55.5)	< 0.001	ABCDEFG---
	1000–1999 births annually	7	31,058	41.3 (32.6–56.2)		
	2000–2999 births annually	9	63,921	40.3 (37.1–47.1)		
	≥3000 births annually	8	110,283	40.3 (32.3–49.1)		
	University hospitals	8	111,631	40.4 (38.0–45.0)		
	All hospitals	38	330,913	40.6 (32.3–56.2)		
Robson 1 and 3 *n* = 219,961	< 1000 births annually	6	9592	42.3 (36.1–56.8)	< 0.001	ABCDEFG-IJ
	1000–1999 births annually	7	21,263	38.6 (28.3–51.2)		
	2000–2999 births annually	9	43,294	36.2 (29.8–42.2)		
	≥3000 births annually	8	74,207	36.4 (27.3–45.7)		
	University hospitals	8	71,605	34.5 (28.9–46.6)		
	All hospitals	38	219,961	36.2 (27.3–56.8)		
Robson 1 *n* = 98,442	< 1000 births annually	6	4047	46.6 (41.4–61.8)	< 0.001	ABCD--G--J
	1000–1999 births annually	7	9272	41.9 (33.9–52.2)		
	2000–2999 births annually	9	19,005	40.6 (33.6–46.3)		
	≥3000 births annually	8	33,931	41.7 (33.8–46.9)		
	University hospitals	8	32,187	39.8 (33.6–46.9)		
	All hospitals	38	98,442	41.1 (33.6–61.8)		
Robson 3 *n* = 121,519	< 1000 births annually	6	5545	39.1 (31.9–53.1)	< 0.001	ABCDEFG-IJ
	1000–1999 births annually	7	11,991	36.0 (23.0–50.5)		
	2000–2999 births annually	9	24,289	32.7 (25.3–41.0)		
	≥3000 births annually	8	40,276	31.9 (20.8–44.7)		
	University hospitals	8	39,418	30.2 (24.6–45.1)		
	All hospitals	38	121,519	32.3 (20.8–53.1)		

^a^ Prevalence of amniotomy in each hospital category

^b^ Pearson chi-square test (the group all hospitals are not included)

^c^ Pairwise comparisons using the Pearson chi-square test with Bonferroni corrected *p*-values (< 0.005); significant differences are presented as: A = 1 ≠ 2, B = 1 ≠ 3, C = 1 ≠ 4, D = 1 ≠ 5, E = 2 ≠ 3, F = 2 ≠ 4, G = 2 ≠ 5, H = 3 ≠ 4, I = 3 ≠ 5, J = 4 ≠ 5

## Discussion

To the best of our knowledge, this is the first nationwide register-based study describing the prevalence of amniotomy in Sweden. We found that almost half of the women giving birth in Sweden during the years 2017–2020 underwent amniotomy. The prevalence remained the same for all births during the study period, but a decrease in amniotomy was seen in Robson group 1 and Robson group 3. Variations in the prevalence between hospitals were observed.

Our findings confirm amniotomy to be one of the most commonly used labor interventions in modern obstetric practice [1]. We found that Robson group 1 was more likely to receive amniotomy compared to Robson group 3. A Chinese study by Gu et al. (2020) had a similar finding to our result [2]. However, the opposite emerged in German and Dutch studies, where multiparous women received amniotomy more often than nulliparous [3, 4]. Nulliparous women generally have longer labors and more often labor dystocia, compared to multiparous [23]. As labor dystocia is the primary indication for labor interventions, higher rates of amniotomy in nulliparous are reasonable. Current research [24–26] aims to reevaluate the “normal” labor progression for the parturient of today, and also to identify how slow is too slow. Having greater patience with labor progression in the first stage of labor, as proposed by several studies [6, 24–26], would certainly call for fewer amniotomies. According to the WHO (2018), health care professionals should support each individual woman in spontaneous labor, by not performing labor interventions with the intention to shorten the duration of labor, provided the condition of the mother and baby is reassuring, and the expected duration of labor is within the recommended limits [6]. Moreover, in spite of the common use of amniotomy to prevent long labors in clinical practice, there is no clear evidence that the potential benefits outweigh the potential harms [1, 6].

During the study period, the prevalence of amniotomy for all births was invariant; thus, for both Robson group 1 and Robson group 3, the prevalence decreased. During the last two decades, the rates of induced labors have been increasing in Sweden, from 10% in 2000 to 27% in 2020 [27]. As amniotomy is regularly used to induce labor, the unchanged prevalence for all births, despite the decrease in the large groups of Robson group 1 and 3, could be explained by the increased rates of induced labors in Sweden. The decrease in the prevalence of amniotomy for Robson groups 1 and 3 may be an effect of the Cochrane-review by Smyth et al. published in 2013, with the clear message to use amniotomy only when indicated, and not routinely [1]. The fact that several other important sources have also called for action to reduce the inappropriate use of labor interventions [5–7, 15] could also have affected the prevalence for women in spontaneous labor. According to Brownlee et al. (2017), there is strong evidence for a widespread overuse of medical services in many countries [8]. Our result, namely that the prevalence of amniotomy for women in spontaneous labor in Sweden indicates a decrease, should be seen as positive.

In our previous study, midwives working at an University hospital experienced that amniotomy was performed due to a constant high work load, therefore we used stratification in order to investigate this issue [28]. Our results in the present study show variations in the prevalence of amniotomy between Swedish hospitals. However, the University hospitals had the lowest prevalence of amniotomy for Robson group 1 and 3. Regional and national variations regarding the prevalence of childbirth interventions have previously been reported in other studies [7, 29, 30]. It has been suggested that variation in the prevalence of interventions indicates an over- or underuse [4, 8, 29]. A first step in addressing an inappropriate use of labor interventions is to explore regional variations in the prevalence within the country [8]. It is of importance to acknowledge that amniotomy is performed for several reasons, occasionally laboring women even request it, as described in our previous study [28]. This fact may contribute to differences in the prevalence between hospitals. Diverse cultures within the working environment at the hospitals could also affect rates of interventions [31]. In a recent Norwegian cohort study by Gjærum et al. (2022), the association between cervical dilatation at hospital admission, and mode of delivery, and rates of intrapartum interventions, was investigated. The authors found that women admitted at cervical dilation of ≥4 cm had a higher chance of spontaneous delivery compared to women admitted at < 4 cm cervical dilatation. Intrapartum interventions such as augmentation with Oxytocin and epidural analgesia were higher for women admitted at < 4 cm cervical dilatation. However, for amniotomy, no differences in rates were observed in the two groups [32]. Unfortunately, the data of the present study included no information on time, nor cervical dilatation, for admission to the hospital,

A major strength of this study is the nationwide register-based design and an almost complete coverage of women giving birth in Sweden. There are some limitations with this study. First, the Swedish Pregnancy Register has no information on the indications for amniotomy, nor the timing of amniotomy in relation to cervical dilatation. This information would have been of importance to understand the varying prevalence between the hospitals. Labor interventions can be effective in preventing or treating complications; however, if used untimely they can instead cause complications [7]. Second, the Robson Classification system was used to identify women with spontaneous labor. A limitation with the Robson Classification system is that it does not fully account for differences in the patient population, such as BMI, age, and history of obstetric complications. This implies that part of the differences in the prevalence between the hospitals could be caused by differences in maternal characteristics. Third, data on prevalence in 2020 were only available for the first half of the year. This was handled by multiplying the prevalence of January to June with two. The same calculations were tested for years with complete data, and the calculations corresponded well to the actual annual prevalence. Potential avenues for future research include indications and complications of amniotomy.

## Conclusions

Amniotomy is a commonly used labor intervention in Sweden, as almost half of the laboring women received amniotomy. The prevalence remained unchanged for all births during the years 2017–2020, but a decrease was seen for women with spontaneous onset of labor. Variations in the prevalence of amniotomy between hospitals were observed, this result may indicate a potential for fewer amniotomies in Swedish childbirth care. Midwives and obstetricians working with labors are encouraged to use amniotomy only when indicated.

## Data Availability

The data that support the findings of this study are available from the Swedish Pregnancy Registry, but restrictions apply to the availability of these data, which were used under license for the current study, and so are not publicly available. Data are however available from the authors upon reasonable request and with permission of the Swedish Pregnancy Registry.

## Abbreviations

BMI: Body Mass Index
CTG: Cardiotocography
WHO: World Health Organization
